# Bacterial RuBisCO Is Required for Efficient *Bradyrhizobium*/*Aeschynomene* Symbiosis

**DOI:** 10.1371/journal.pone.0021900

**Published:** 2011-07-05

**Authors:** Benjamin Gourion, Nathanaël Delmotte, Katia Bonaldi, Nico Nouwen, Julia A. Vorholt, Eric Giraud

**Affiliations:** 1 Laboratoire des Symbioses Tropicales et Méditerranéennes, Institut de Recherche pour le Développement/SupAgro/Institut National de la Recherche Agronomique/Université Montpellier 2/Centre de Coopération Internationale en Recherche Agronomique pour le Développement, Montpellier, France; 2 Institute of Microbiology, Eidgenössische Technische Hochschule Zurich, Zurich, Switzerland; University of Glasgow, United Kingdom

## Abstract

Rhizobia and legume plants establish symbiotic associations resulting in the formation of organs specialized in nitrogen fixation. In such organs, termed nodules, bacteria differentiate into bacteroids which convert atmospheric nitrogen and supply the plant with organic nitrogen. As a counterpart, bacteroids receive carbon substrates from the plant. This rather simple model of metabolite exchange underlies symbiosis but does not describe the complexity of bacteroids' central metabolism. A previous study using the tropical symbiotic model *Aeschynomene indica*/photosynthetic *Bradyrhizobium* sp. ORS278 suggested a role of the bacterial Calvin cycle during the symbiotic process. Herein we investigated the role of two RuBisCO gene clusters of *Bradyrhizobium* sp. ORS278 during symbiosis. Using gene reporter fusion strains, we showed that *cbbL1* but not the paralogous *cbbL2* is expressed during symbiosis. Congruently, CbbL1 was detected in bacteroids by proteome analysis. The importance of CbbL1 for symbiotic nitrogen fixation was proven by a reverse genetic approach. Interestingly, despite its symbiotic nitrogen fixation defect, the *cbbL1* mutant was not affected in nitrogen fixation activity under free living state. This study demonstrates a critical role for bacterial RuBisCO during a rhizobia/legume symbiotic interaction.

## Introduction

The Calvin Benson Bassham cycle (CBB cycle), also termed reductive pentose phosphate pathway, is the quantitatively most important mechanism of autotrophic CO_2_ fixation in nature [1]. It's key enzyme ribulose 1,5 bis-phosphate Carboxylase Oxygenase, RuBisCO catalyzes the formation of two molecules of 3 phosphoglycerate from ribulose bisphosphate and CO_2_ and has been subject to extensive studies since its discovery [2]. Besides fundamental interest in carbon assimilation, research in this field is also motivated by the objective to improve crop yield and by the capacity of CO_2_ fixation pathways to transform the greenhouse gas [3] [4].

Whereas in plants and other autotrophic organisms, the CBB cycle plays an essential role in biomass production, the presence of a functional CBB cycle in heterotrophic organisms is less obvious. The operation of the calvin cycle is costly and consumes both ATP and reducing power (3 ATP and 4 reducing equivalents per CO_2_ assimilated). It thus appears paradoxal that organic carbon compounds could be oxidized to CO_2_ at the same time that CO_2_ is assimilated by the cell. The phenomenon of substantial carbon fixation in the presence of multicarbon compounds was observed earlier and an alternative role of the CBB cycle in some microorganisms suggested that consists in redox cofactor balancing [5], [6], [7], [8], [9].

*Sinorhizobium meliloti*, *Bradyrhizobium japonicum* and the photosynthetic *Bradyrhizobium* strain BTAi1 are known to assimilate CO_2_ *via* the CBB cycle [10], [11], [12]. These microorganisms belong to the polyphyletic rhizobial group. Rhizobia are soil bacteria able to form symbiotic interactions with legume plants. During these symbiotic interactions, plants develop new organs on their root system. In these organs, called nodules, bacteria reside intracellularly and their metabolism is drastically modified as it is largely dedicated to the reduction of atmospheric nitrogen that is made available to the plant host. In return bacteria receive organic carbon in the form of dicarboxilic acids from the plant. This basic assimilated carbon versus nitrogen exchange is the basis of the symbiotic relationship between both partners [13] but the metabolites exchanges seems much more complex [14].

Photosynthetic bradyrhizobia induce and colonize nodules on semi-aquatic legume plants belonging to the *Aeschynomene* genus [15]. Beyond their photosynthetic trait, bacteria involved in these interactions present other atypical aspects compared to classical rhizobia. They can fix nitrogen in the free living state, an ability restricted among rhizobia to photosynthetic bradyrhizobia and to *Azorhizobium caulinodans* [16], [17]. Furthermore, photosynthetic bradyrhizobia lack the *nodABC* genes, indicating that these bacteria interact with their legume host according to a new molecular mechanism independent of the long-time considered universal Nod factors [18]. Finally, in accordance with the semi-aquatic nature of their *Aeschynomene* host, they distinguish from classical rhizobia by their adaptation to the aquatic environment beyond the classical rhizobial habitats (rhizo-, phyllo-sphere, soil and root nodules). As a consequence, photosynthetic bradyrhizobia nodulate not only the roots but also the stems of *Aeschynomene* plants when the latter are subjected to immersion. This capacity to colonize various environments (stem and root nodules, plant surfaces, soil, and flooded area) might rely on versatile metabolic skills. As an example, photosynthetic capacities of the model *Bradyrhizobium sp*. ORS278 were shown to be important for stem nodulation but not root nodulation [19].

**Figure 1:**
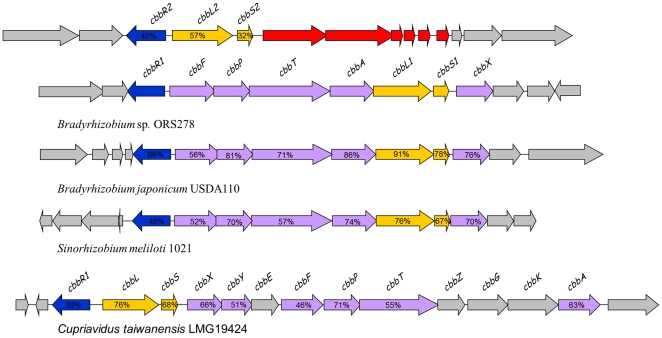
Genetic organization of RuBisCO 1 (encoded by BRADO1659 and BRADO1660) and RuBisCO 2 (encoded by BRADO2274 and BRADO2275) loci of *Bradyrhizobium* sp. ORS278 and corresponding synthenic regions of RuBisCO 1 in other rhizobial models. LysR type regulator are represented in blue, RuBisCO genes in yellow, other CBB cycle genes in purple and carboxysome genes in red. Amino-acid identity level is represented as a percentage for the genes found in synteny.

In a previous study aiming at identifying ORS278 mutants altered in the symbiotic process, four Tn5 insertional mutants potentially affected in the CBB cycle were described as altered in their symbiotic nitrogen fixation capacity (Fix^-^ phenotype) [20]. ORS278 genome harbours two RuBisCO gene clusters [18] encoding for proteins which belong to the form I RuBisCO of heterodimeric proteins containing 8 large and 8 small subunits. Figure 1 describes the genetic organization of the RuBisCO genes clusters. The two enzymes, called above RubisCO 1 and 2, are encoded by *cbbL1*/*cbbS1* and *cbbL2*/*cbbS2* respectively. Genes corresponding to RuBisCO 1 are located in a locus containing most of the genes required for the reductive pentose phosphate pathway (Figure 1). RuBisCO 2 gene region harbours typical traits of a horizontally acquired gene cluster [18]. Downstream of *cbbLS2*, genes involved in carboxysome formation are found. Carboxysomes are proteic microcompartments found mainly in Cyanobacteria and some chemoautotrophs. These organelle-like structures are responsible for CO_2_ concentration around the RuBisCO enzyme [21], [22]. A gene coding for a CbbR regulator is located upstream of *cbbLS2* and divergently transcribed. Such a regulator is also found upstream of RuBisCO 1 gene cluster (Figure 1). The existence of two RuBisCO genes, both associated with a regulator, suggests that ORS278 might differentially use one or the other enzyme depending on environmental conditions.

Herein we investigated the importance of RuBisCO 1 and 2 during the symbiotic process and demonstrate the contribution of RuBisCO 1 to symbiosis.

## Results

### Bradyrhizobium spp. ORS278 genome houses two physically and phylogenetically distant RuBisCO gene clusters

**Figure 2:**
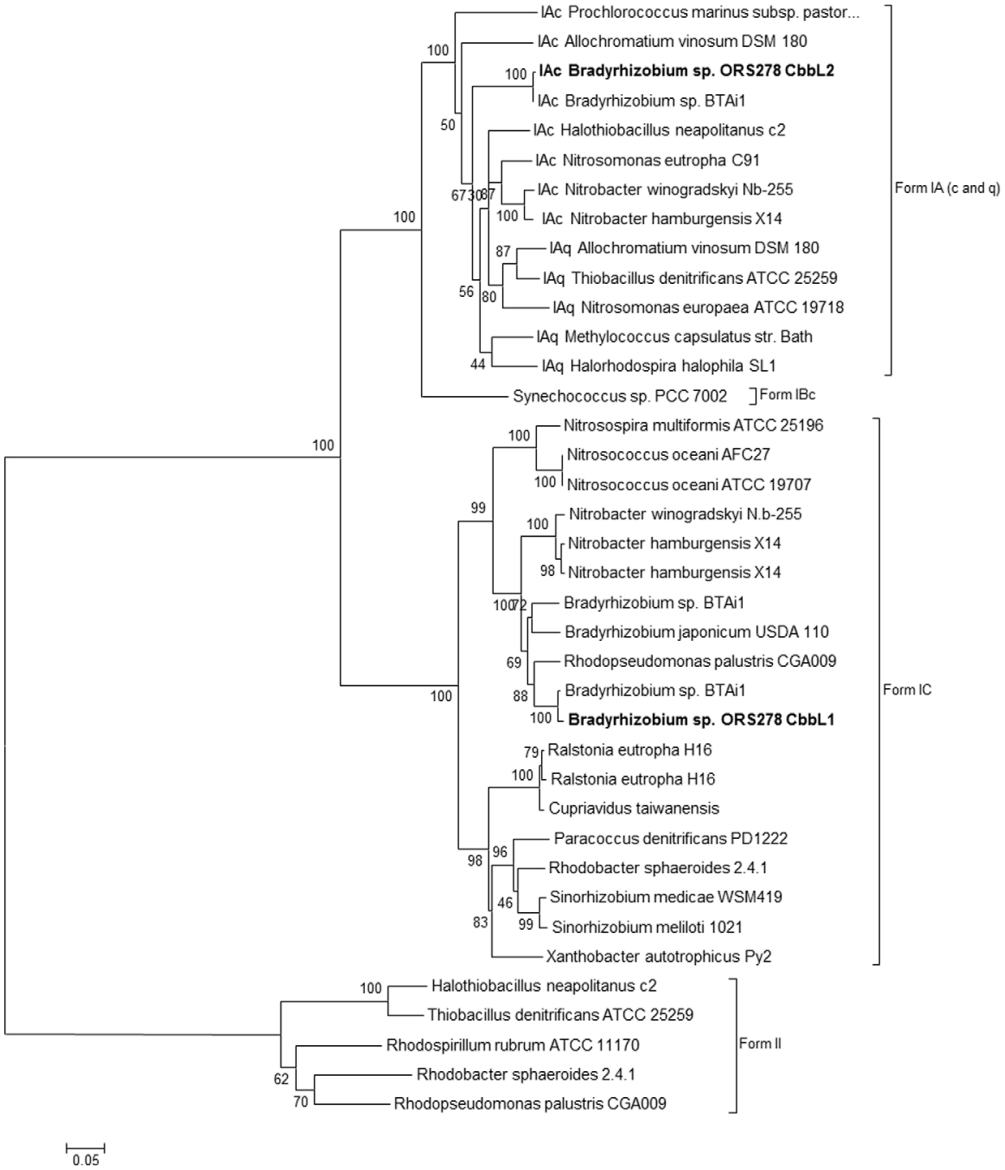
Evolutionary relationships of RuBisCO large subunit sequences. Neighbour joining method was used. Bootstrap support values (10000 replicates with Mega4) are provided as percentage at the corresponding nodes. CbbL1 and CbbL2 proteins from ORS278 strain are highlighted in bold characters. For RuBisCO IA members, the RuBisCO subclasses IAc and IAq is indicated before bacterial name.

As mentioned above, the two predicted ORS278 CbbL proteins belong to the group of Type I RuBisCO enzymes, nevertheless they exhibit a sequence identity of only 57%. Neighbour joining analysis performed on various procaryotic RuBisCO large subunit sequences showed that the two ORS278 genes belong to two distinct clades (Figure 2). RuBisCO 1 belongs to the IC RuBisCO class, a group related to medium to high CO_2_ environments [23]. Beyond the CbbL1 closest ortholog in BTAi1 (99% of identity), predicted proteins are well conserved in some non-photosynthetic rhizobia such as *Sinorhizobium meliloti* with an identity level of 76% [24] or *B. japonicum* (91% identity). In these three strains, the genomic context is well conserved and the whole gene clusters exhibit a perfect microsyntheny (Figure 1). A CbbL1 homologue (76% of identity) is also present in the phyllogeneticaly distant beta-rhizobium *Cupriavidus taiwanensis* [25]. RuBisCO 2 belongs to the IAc RuBisCO class, which harbors orthologs from some other Alphaproteobacteria such as *Nitrobacter* strains, Beta- and Gammaproteobacteria as well as Cyanobacteria, this class is related to low CO_2_ environments [23]. The only known rhizobial ortholog in the IAc clade is *Bradyrhizobium* sp. BTAi1 (>99% of identity).

### Only *cbbL1* is expressed in planta

**Figure 3:**
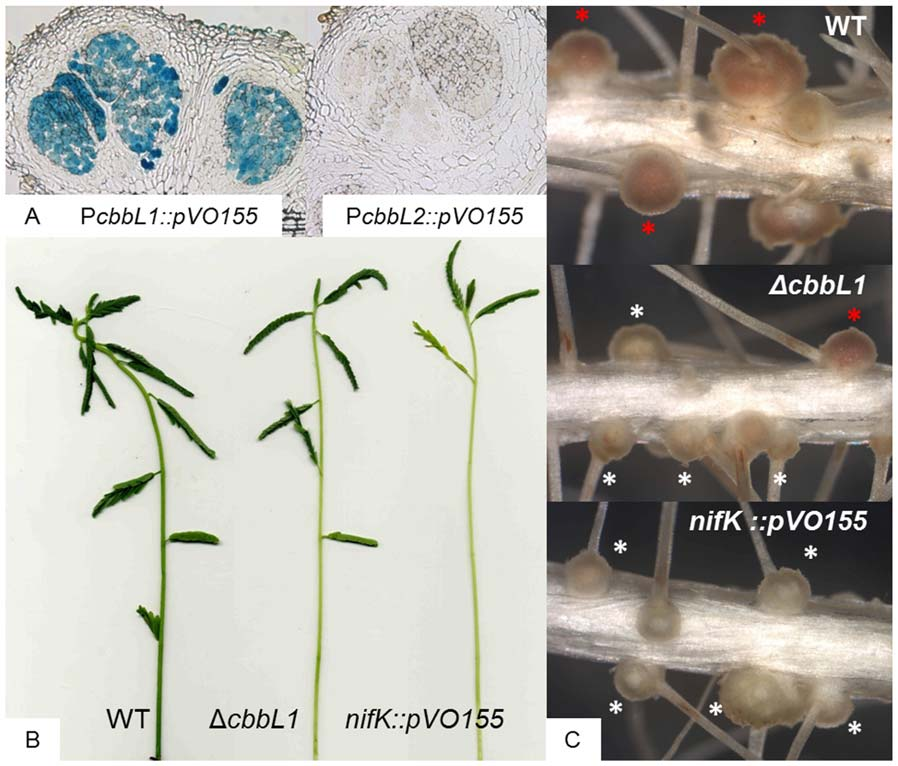
*cbbL1* and *cbbL2* expression and symbiotic phenotypes of the *cbbL1* mutant compared with the WT-strain and the *nifK* mutant on *A. indica*. A. Transcriptional activity of the *cbbL1* and *cbbL2* putative promoter regions fused to *gusA* gene revealed on 40 µm nodule sections stained with X-gluc. B. Aerial part and C. root systems of plants inoculated with *Bradyrhizobium* sp. ORS278 and *cbbL1* and *nifK* derivative strains, white and red asterisk indicate fix minus like and WT-like nodules respectively.

**Figure 4:**
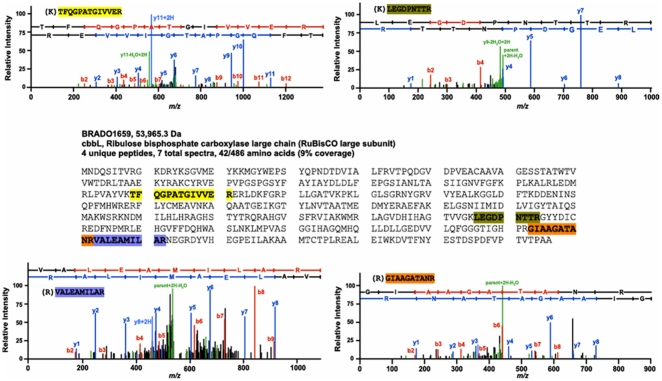
Identification of CbbL1 (BRADO1659) peptides in bacteroids protein extract by tandem mass spectrometry and database search.

In order to determine any potential contribution of RuBisCO 1 or 2 during the symbiotic process, reporter strains carrying *gus* fusion downstream of the predicted promoter region of the RuBisCO gene clusters were constructed. *A. indica* nodules of different age induced by these strains (5, 7, 10, 14, 21 days post-inoculation) were sliced into thin layers, stained with X-Gluc and reporter gene expression was monitored by standard light microscopy. No detectable activity of *gus* reporter fusion was revealed for the *cbbL2* promoter region. In contrast, the 5′ region of the *cbbL1* gene cluster displayed a transcriptional activity detectable at every stages of nodule development examined even at five dpi when first young nodules appeared. This expression did not seem to diminish in mature nodules (Figure 3A corresponds to 14 dpi nodule). In order to determine if CbbL1 protein is produced in nodules, a proteomics analysis was performed on bacteroids isolated from *A. indica* 14 dpi nodules. Protein extract was separated by SDS-PAGE and a gel section corresponding to CbbL1 and 2 predicted molecular weight was analysed by trypsin digestion followed by LC-MS/MS. Fragmentation profiles allowed the identification of four peptides from CbbL1 (Figure 4) but none of CbbL2.

### *cbbL1* is required for efficient symbiotic nitrogen fixation

**Figure 5:**
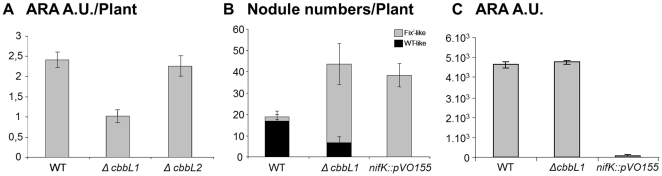
RuBisCO 1 mutant displays typical traits of Fix^-^ mutant on *A. indica* but is able to reduce acetylene *in vitro*. A. Symbiotic acetylene reduction assays: *A. indica* plants were inoculated with the indicated *Bradyrhizobium* sp. ORS278 strain one week after germination. Acetylene reduction activities were measured on individual plants two weeks after inoculation. B. Nodule numbers induced by the indicated strain on *A. indica* were determined two weeks after infection. C. Acetylene reduction activities were measured during growth on BNM medium supplemented with oxo-glutarate. Error bars represent standard deviations.

In order to evaluate the importance of RuBisCO 1 or 2 during the symbiotic process, mutant strains harbouring a deletion in one of the two genes coding for the large RuBisCO subunit CbbL were constructed and inoculated on *A. indica*. To prevent any effect of bacterial photosynthesis and mimic natural conditions, plants were root inoculated and roots systems were maintained in the darkness. Two weeks after inoculation, acetylene reduction assays were conducted to evaluate nitrogenase activity of plants housing the different strains. Whereas no significant defect was observed for plants inoculated with the *cbbL2* mutant, plants inoculated with the *cbbL1* mutants exhibited a nitrogenase activity defect ranging from 30 to 50% to that of the wild type (WT) strain (Figure 5A). This result was consistent with phenotypic observations of three week old plants which revealed that plants inoculated with a *cbbL1* mutant but not those inoculated with a *cbbL2* mutant exhibited typical nitrogen starvation symptoms such as foliage chlorosis or stem thinning (Figure 3B). Symptom intensity did not reach those observed on plants inoculated with a *nifK* mutant which was altered in the nitrogenase enzyme itself and therefore displayed a strict Fix^-^ phenotype.

### The *cbbL1* mutant induces more nodules which are heterogenic in phenotype

Root systems of plants inoculated with the *cbbL1* mutant harboured two times more nodules than those inoculated with the WT strain (Figure 5B) and was thus in the same range as observed upon inoculation of a *nifK* mutant. On every root system, *cbbL1* induced more than 80% of nodules with a yellow colour typically indicative of nitrogen fixation deficiency, the remaining being WT-like reddish nodules (Figure 3C, 5B). In order to determine if a secondary mutation occurred in *cbbL1* mutants present in WT-like nodules, bacteria were rescued from these nodules. After cultivation, mutation in *cbbL1* was confirmed by PCR and these *cbbL1* mutants derived from WT-like nodules were inoculated on new plants. The resulting root systems displayed also a mixture of WT-like and yellowish nodules. This result demonstrated the heritable nature of the heterogenic phenotype and excluded the possibility of secondary mutations in *cbbL1* mutants forming WT-like nodules.

### *cbbL1* is not required for in vitro nitrogen fixation

In order to determine whether the symbiotic defect observed with the *cbbL1* mutant was directly related to nitrogen fixation capacity, we investigated the bacterial capacity to fix nitrogen under free-living conditions. Using succinate, malate or 2-oxoglutarate as a sole source of carbon and energy, no significant nitrogenase activity defect was revealed by acetylene reduction assays (Figure 5C and not shown) demonstrating the absence of direct correlation between the presence of RuBisCO 1 and the capacity to fix nitrogen.

## Discussion

The presence of an active RuBisCO has been reported in several rhizobial strains including photosynthetic and non-photosynthetic rhizobium; however, to our knowledge, so far no role of this enzyme during the symbiotic process has been reported. In this study, we demonstrate that one of the two RuBisCO enzymes identified in the photosynthetic *Brayrhizobium* strain (ORS278) is required for an efficient symbiosis with *A indica* plants. First, we showed expression of the *cbbL1* but not *cbbL2* gene during the symbiotic process and second, we observed a reduced symbiotic nitrogenase activity level of plants inoculated with *cbbL1* mutant leading to typical nitrogen starvation symptoms such as foliage chlorosis and stem thinning. *cbbL1* interacting plants display nitrogen starvation symptoms weaker than plants housing nitrogenase minus mutants. This intermediary phenotype can be explained by the heterogeneity of the induced nodules. Indeed, *cbbL1* induced a mixture of WT-like, reddish, and fix minus-like, yellowish, nodules on the same plants. Yellowish nodules are likely nitrogen fixation deficient whereas the WT-like nodules probably fix and transfer nitrogen to the host, partially compensating nitrogen deprivation. This heterogeneous phenotype and *cbbL1* mutant capacity to fix nitrogen *in vitro* indicate that *cbbL1* is not essential for nitrogen fixation. Thus, the importance of RuBisCO 1 during the symbiotic process is probably not related to mature bacteroid metabolism but to an early step of the symbiosis.

**Figure 6:**
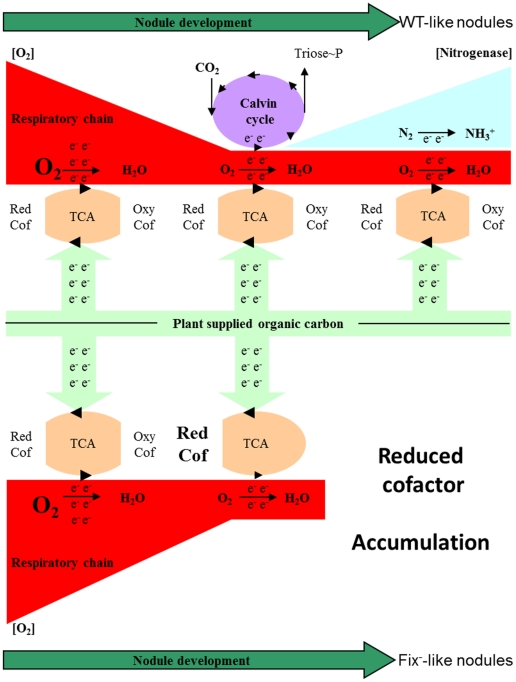
Hypothetical model of ORS278 central metabolism during the symbiotic process. Upper part of the figure represents the WT and WT-like situation: Plant supplied organic carbon to bacteria which use it via the tricarboxylic acid cycle (TCA) resulting in the production of reduced cofactors (Red Cof). Reduced cofactors are totally oxidized by the respiratory chain at the early stage of nodule formation, then oxygen concentration drops and Calvin cycle drives the electron excess to CO_2_. Once nitrogenase synthesis has started, electron excess is consumed by the nitrogen fixation process. Lower part of the figure represents the situation with a *cbbL1* mutant leading to a Fix–like nodule: electron excess cannot be drive to CO_2_ therefore, reduced cofactors accumulate and disturbed bacterial metabolism preventing normal nodule development.

Results presented here were obtained with plant root systems maintained in the darkness. Under this condition bacteria produce energy by oxidation of reduced carbon compounds supplied by the plant. In this context, the operation of the CBB cycle as a mean to produce biomass seems futile since reduced carbon is provided by the plant. In some photosynthetic bacteria, the CBB cycle plays an essential role during growth on reduced carbon substrates in the absence of oxygen [7], [26]. In these bacteria, the CBB cycle acts as an electron sink driving the cell electron excess to CO_2_ and thus oxidizing reduced cofactor and contributing in a significant way to equilibrate the redox state of the cell [6]. Interestingly, in *Rhodospirillum rubrum* and *Rhodobacter sphaeroides*, a RuBisCO mutant which is unable to grow photoheterotrophically, can be rescued by acquiring secondary mutation de-repressing nitrogen fixation genes [27]. Joshi and Tabita (1996) and more recently McKinley and Harwood (2010) explained these observations by the high reductant requirement of nitrogenase which, like the Calvin cycle, can play an electron sink role. We propose ORS278 strain uses the Calvin cycle for this purpose at an early stage of the symbiotic process: when oxygen concentration in the nodule has already significantly decreased and before the establishment of functional nitrogenase. At such a step, reduced cofactors pool produced primarily by the Krebs cycle might not be balanced and the CBB cycle might fulfil this function which, in developed bacteroids, is fulfilled by nitrogenase activity (Figure 6). The dynamical dimension of this hypothetical model offers an explanation to the observed WT-like nodules induced by *cbbL1* mutant. Indeed, oxygen concentration decreasing rate in the nodule and nitrogenase production might be influenced by diverse parameters and we may consider the possibility that in some nodules oxygen concentration decreases more slowly and nitrogenase production starts more vigorously than on the average nodule which would not allowed the accumulation of reductant making useless the Calvin cycle.

More work will be required to identify a role for RuBisCO 2 as the *cbbL2* mutant did not display any phenotype during the symbiotic process or nitrogen fixation *in vitro*. Transcriptional activity from *cbbL2* promoter was not detected during growth on rich or minimal medium using various carbon compounds. It is possible that ORS278 strain uses this enzyme under yet unidentified environmental conditions or that this cluster is not yet functional in this host strain due to its recent acquisition.

## Materials and Methods

### Phyllogenetic analysis

RuBisCO large sub-unit sequences from various bacteria YP_001735042.1 (*Synechococcus* sp. PCC 7002); NP_892668.1 (*Prochlorococcus* marinus subsp. *pastoris* str. CCMP1986); YP_001416820.1 (*Xanthobacter autotrophicus* Py2); YP_001203774.1 and YP_001204346.1 (*Bradyrhizobium* sp. ORS278); YP_002008347.1 (*Cupriavidus taiwanensis*); NP_769225.1 (*Bradyrhizobium japonicum* USDA 110); NP_841943.1 (*Nitrosomonas europaea* ATCC 19718); YP_115143.1 (*Methylococcus capsulatus* str. Bath); NP_946905.1 and NP_949975.1(*Rhodopseudomonas palustris* CGA009); NP_436731.1 (*Sinorhizobium meliloti* 1021); YP_427487.1 (*Rhodospirillum rubrum* ATCC 11170); YP_354363.1; YP_354780.1 (*Rhodobacter sphaeroides* 2.4.1); YP_001236643.1; YP_001238690.1 and YP_001242213.1 (*Bradyrhizobium* sp. BTAi1); YP_316382.1 and YP_316396.1 (*Thiobacillus denitrificans* ATCC 25259); YP_915492.1 (*Paracoccus denitrificans* PD1222); YP_571545.1, YP_571759.1 and YP_578935.1 (*Nitrobacter hamburgensis* X14); YP_318598.1; YP_319531.1 (*Nitrobacter winogradskyi* Nb-255); YP_342389.1 (*Nitrosococcus oceani* ATCC 19707); YP_411385.1 (*Nitrosospira multiformis* ATCC 25196); YP_747036.1 (*Nitrosomonas eutropha* C91); YP_001002624.1 (*Halorhodospira halophila* SL1); YP_001312667.1 (*Sinorhizobium medicae* WSM419); NP_943062.1 and YP_840914.1 (*Ralstonia eutropha* H16); ZP_05049432.1 (*Nitrosococcus oceani* AFC27); YP_003262812.1 and YP_003262978.1 (*Halothiobacillus neapolitanus* c2); YP_003443332.1; YP_003444691.1 (*Allochromatium vinosum* DSM 180)) were aligned using clustalW multiple alignment [28] with a bootstrap of 10000 replicates. Phyllogenetic tree was produced using Mega 4 [29].

### Bacterial Strains and Growth Conditions

*Bradyrhizobium* sp. strain ORS278 [30], and derivatives Δ*cbbL1*, Δ*cbbL2*, *PcbbL1::pVO155* and *PcbbL2::pVO155* constructed in this work were grown in a modified YM medium (pH 6.8) as described in [19]. Strains were grown aerobically in a gyratory shaker (170 rpm) at 37°C. Standard methods were used for *Escherichia coli* growth in Luria–Bertani (LB) medium supplemented with the appropriate antibiotics.

### Strains constructions

In order to construct mutant strains deleted in Brado1659 and Brado2274, DNA fragments were amplify from ORS278 genomic DNA using primer pairs Brado1659F (5′- TGCAGGACATGTTCAATCAGTTC-3′) /Brado1659R (5′-GGCCGGTTCACGATGAAGGACAG-3′) and Brado2274F (5′- GAGTTGTTTGCACTGCATCGAAAC-3′) /Brado2274R (5′-CACAGGAGCCGGTGAAGCAATC-3′). Resulting fragments were cloned in pGEMT easy and transformed in thermocompetent XL2-blue. Brado2274 and Brado1659 DNA fragments were excised from pGEMT using ApaI/SpeI and NotI respectively and cloned into pJQ200uc1 [31] (Brado 1659) and pJQ200sk [31](Brado 2274) opened with NotI and ApaI/SpeI respectively resulted in pBG1659 and pBG2274. Ligation products were transformed into XL2-Blue cells. Once check by restriction profiles, resulting plasmids were digested with XhoI/SalI (pBGBrado2274) and SalI (pBGBrado1659) and kanamycin resistance cassette excised from pKOK5 [32] using SalI was cloned into linearized vectors. Plasmids were transferred in *E. coli* S17-1 [33] by electroporation prior to mating with ORS278. Transconjugants were selected using nalidixic acid and kanamycin. Then transconjugants were replicated on sucrose medium containing kanamycin. Resulting colonies were check by PCR amplification of cassette flanking regions.

*nifK* mutant: Brado5438R/Brado5438F (5′-TCTAGACGTGTCGAACACATTCGAGTTGTC-3′/5′GTGTCGACCTGCATGGCGGAAGTC-3′) were used to amplify the central part of *nifK* open reading frame. DNA fragment was cloned into pGEMT easy and ligation product was transformed into XL2-Blue. After verification, insert was excise using SalI/XbaI and cloned into SalI/XbaI linearised pVO155 [34]. Resulting plasmid was successively transferred into XL1-Blue and S17-1. Resulting S17-1 strain was used to introduce the construction in ORS278 by mating.

Reporter strains: putative *cbbL1* and *cbbL2* promoter regions were PCR amplified using Brado1655PromF/Brado1655PromR (5′-CGATGATGATGTGCGGATGCTTG-3′ and 5′-GTGTCTAGAGATGATGATCCATCGGGTGATTG-3′) and Brado2274PromF/Brado2274PromR (5′- GAGGTCGACCTGATTGTCGGCCAAG-3′ and 5′-CCTTCTAGACGATCTTGAAGACCGCGAGAAG-3′) primer pairs.

DNA fragments were cloned in pGEMT easy resulting in PcbbL1_pGEMt and PcbbL2_pGEMt. SalI/XbaI insert were then excised, purified and ligated in pVO155 suicide vector [34] linearised with SalI/XbaI. Ligation products were introduced in XL2-Blue and S17-1 successively and finally transferred to ORS278 by mating.

### Plant cultivations and inoculation

*A. indica* seeds were surface sterilized by immersion in sulphuric acid under shaking during 45 minutes. Seeds were abundantly washed with sterile distilled water and incubated overnight in sterile water. Seeds were then transferred for two days at 37°C in the darkness on 0.8% agar plate for germination. Plantlets were then transferred on the top of test tubes covered by aluminium paper for hydroponic culture in buffered nodulation medium (BNM) [35]. Plants were grown in closed mini green house in a 28°C growth chamber with a 16-h light and 8-h dark regime and 70% humidity. Seven days after transfer, each seedling was inoculated with one milliliter of cell suspension resulting from a 5 day-old bacterial culture washed in BNM and adjusted to reach an optical density of one at 600 nm.

### Histochemical analysis and microscopy

30-to 40-µm-thick vibratome (VT1000S; Leica, Nanterre, France) sections from fresh nodule samples were observed under bright-field illumination with an optical microscope (PROVIS; Olympus, Rungis, France). To follow glucuronidase activity, nodule thick sections were incubated at 37°C in the darkness in a GUS assay buffer for 4 hours. This reaction buffer was described in [36] and contained 0.075% (wt/vol) of X-Gluc (5-bromo-4-chloro-3-indolyl-â-D-glucuronide) in 0.1 M K/Na phosphate buffer (pH 7.0), 20 mM NaEDTA, 0.02% Triton X-100, 1 mM K-ferricyanide, and 1 mM K-ferrocyanide.

### Proteomics analysis

21 days post inoculation nodules were harvested and immediately flash frozen in liquid nitrogen. Bacteroids were isolated on a sucrose density gradient using a procedure derived from [37] using modifications described in [38] and [39]. Proteins were separated and analysed as described in [40].

### Acetylene reduction assays

*In planta* acetylene reduction assays were conducted as described in [20] with minor modifications. Briefly: one plant was placed into 125 mL glass vials sealed with rubber septa. To avoid overpressure, 12.5 ml of air were removed before injecting the same volume of acetylene. After 3 hours of incubation at room temperature, the ethylene produced was measured by GC-FID using one ml of sampled gas. At least ten plants were used per condition. Plants inoculated with ORS278 strain were used as a reference. *In vitro* acetylene reduction assays were performed as described in [16].
